# Supplementary material for: The interactome of intact mitochondria by cross-linking mass spectrometry provides evidence for coexisting respiratory supercomplexes
Source: Mol Cell Proteomics. 2017 Dec 8;17(2):216–32. doi: 10.1074/mcp.RA117.000470 (PMC5795388; doi:10.1074/mcp.RA117.000470)
Supplement: Supplemental Data [file supp_RA117.000470_133922_0_supp_23978_fzffwf.zip › spectra_annotation/mito_DR_spectra_annotation/156-1-1-1-1-1.pdf]

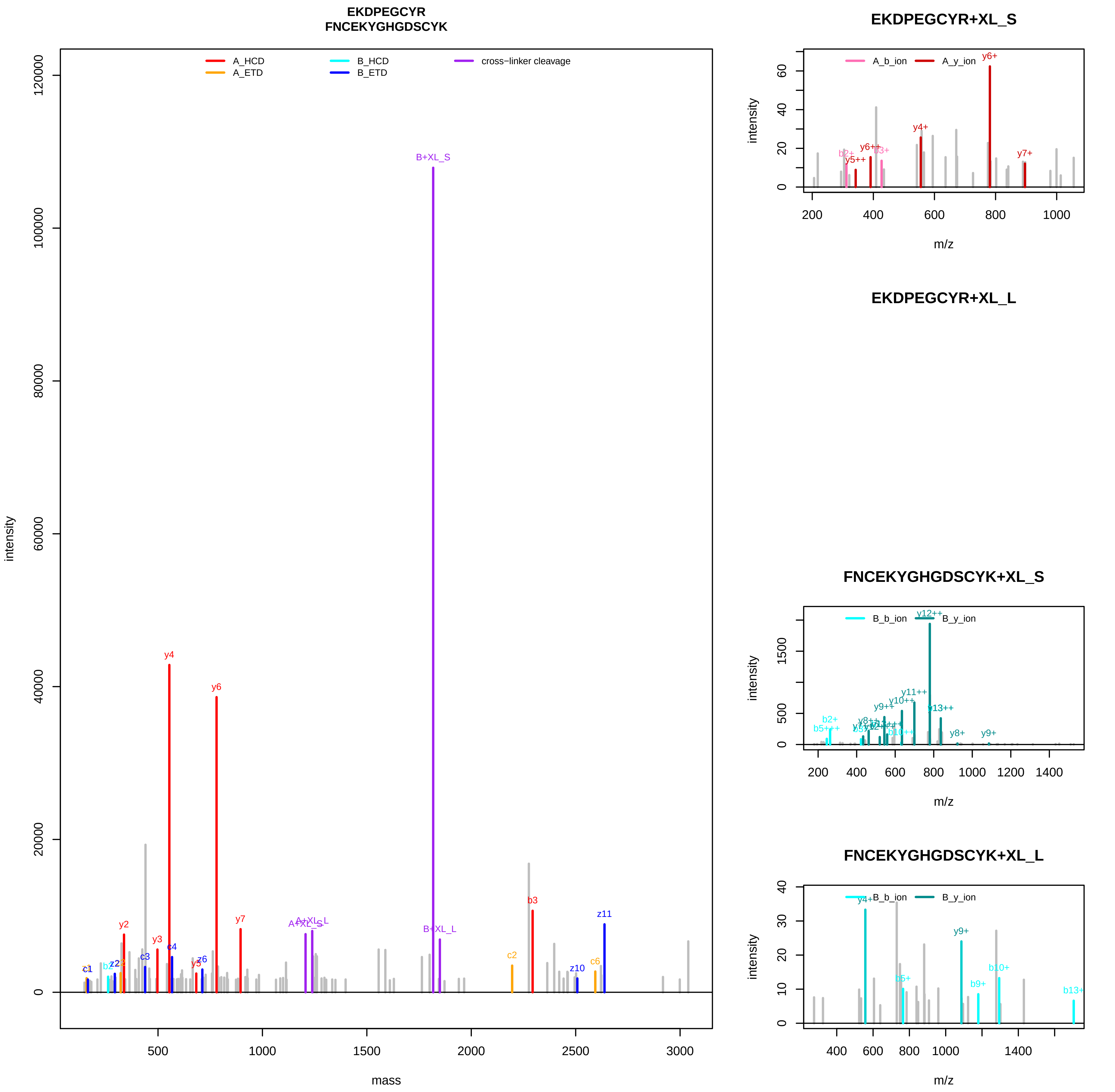
EKDPEGCYR
FNCEKYGHGDSCYK
A_HCD
A_ETD
B_HCD
B_ETD
cross−linker cleavage
B+XL_S
B+XL_L
A+XL_S
A+XL_L
y2
y3
y4
y5
y6
y7
b3
c1
c2
c3
c4
c6
z2
z6
z10
z11
intensity
mass
EKDPEGCYR+XL_S
A_b_ion
A_y_ion
y6+
y4+
y6++
y5++
y7+
b2+
b3+
m/z
EKDPEGCYR+XL_L
FNCEKYGHGDSCYK+XL_S
B_b_ion
B_y_ion
y12++
y11++
y10++
y9++
y13++
y8+
y9+
b2+
b5+++
FNCEKYGHGDSCYK+XL_L
y4+
y9+
b5+
b9+
b10+
b13+
